# Human Umbilical Cord Mesenchymal Stem Cell‐Derived Microvesicles Alleviate Bronchopulmonary Dysplasia in Preterm Rats by Inhibiting Neutrophil Extracellular Trap Formation

**DOI:** 10.1002/pdi3.70065

**Published:** 2026-08-05

**Authors:** Yi Lu, Wen Tan, Xiao Yang, Tingting Luo, Chenghao Mei, Huaqin Bu, Jinhua Ma, Chao Gong, Li Zhong, Ou Zhou, Daiyin Tian

**Affiliations:** ^1^ Ministry of Education Key Laboratory of Child Development and Disorders, Department of Respiratory Medicine, Children's Hospital of Chongqing Medical University National Clinical Research Center for Children and Adolescents' Health and Diseases Chongqing China; ^2^ Chongqing Engineering Research Center of Stem Cell Therapy Chongqing China; ^3^ Department of Pulmonology Children's Hospital of Fudan University National Children's Medical Center Shanghai China

**Keywords:** bronchopulmonary dysplasia, lipopolysaccharide, lung inflammation, mesenchymal stem cells, microvesicles, neutrophil extracellular traps

## Abstract

Among preterm infants, bronchopulmonary dysplasia (BPD) is the most prevalent chronic pulmonary disorder. Its pathogenesis involves a complex interplay between prenatal inflammatory exposure and abnormal immune activation. Neutrophil extracellular traps (NETs) are mediators of tissue damage, but whether they bridge intrauterine inflammation and the subsequent development of BPD is not yet clear. We used a prenatal lipopolysaccharide (LPS)‐exposed rat model to ask two questions: first, whether excessive NETs drive BPD pathogenesis, and second, whether human umbilical cord mesenchymal stem cell‐derived microvesicles (hUCMSC‐MVs) can alleviate lung injury by inhibiting this process. We established a BPD model in preterm rats via prenatal LPS exposure and assessed lung morphometry, neutrophil infiltration, and NETs markers. We then compared the therapeutic effects of hUCMSC‐MVs with NET extrusion (NETosis) inhibitors in vivo and also examined direct neutrophil effects in vitro. Prenatal LPS exposure led to significantly elevated NET markers in both lung tissue and circulation, which correlated with severe alveolar simplification. Neutrophils in this model exhibited a “primed” phenotype with enhanced potential for NETs release. In vivo, hUCMSC‐MVs suppressed NET formation, attenuated neutrophilic inflammation, and restored alveolar structure, matching the efficacy of conventional NETosis inhibitors. In vitro, they were internalized by neutrophils and abrogated LPS‐induced NETosis. These findings point to excessive NETosis as a key mechanistic link between prenatal inflammation and postnatal lung impairment. hUCMSC‐MVs directly inhibit this pathological process and alleviate BPD‐like lung injury. This work advances the mechanistic understanding of BPD and supports hUCMSC‐MVs as a promising cell‐free therapeutic candidate.

## Graphical abstract

Excessive neutrophil extracellular trap (NET) extrusion (NETosis) links prenatal inflammation to bronchopulmonary dysplasia (BPD) pathogenesis. Treatment with human umbilical cord mesenchymal stem cell‐derived microvesicles suppresses NET formation, reduces lung injury, and promotes alveolarization in a preterm rat model, offering a promising cell‐free approach for BPD therapy.

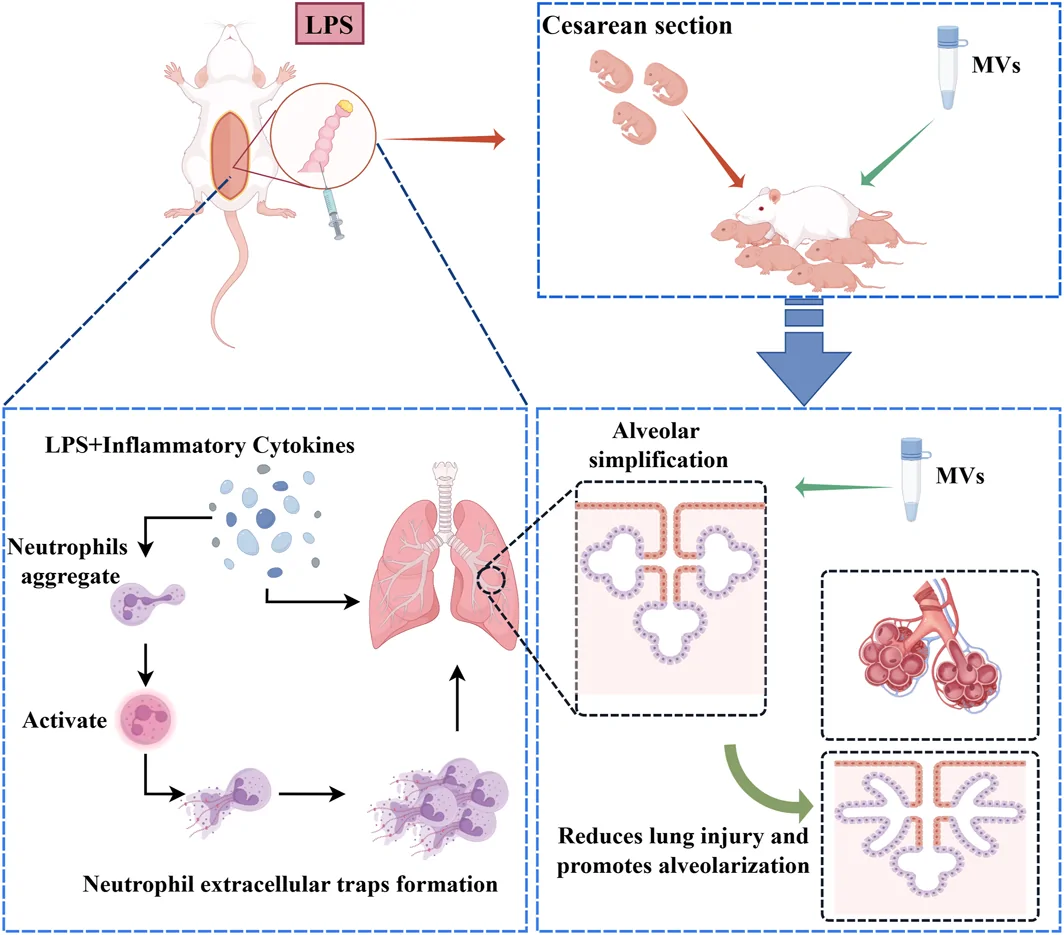

## 1 Introduction

Among preterm infants, bronchopulmonary dysplasia (BPD) represents the most frequently encountered chronic pulmonary disorder, with its occurrence rate escalating markedly as gestational age and birth weight diminish [1, 2]. Despite significant improvements in neonatal intensive care that have boosted survival rates for extremely preterm infants, the occurrence of BPD remains high [3, 4]. This suggests that conventional postnatal influences, such as excessive oxygen exposure and the use of mechanical ventilation alone, cannot fully account for the pathogenesis of BPD [4, 5, 6]. Consequently, research attention has shifted to the prenatal period.

Recent findings suggest that intrauterine infection and inflammation are crucial in the development of BPD [5, 7, 8]. For example, lipopolysaccharide (LPS)‐induced chorioamnionitis is considered a key trigger for abnormal fetal lung development [9, 10]. However, the specific molecular mechanisms linking prenatal inflammatory exposure to postnatal BPD remain poorly elucidated.

Upon activation, neutrophils release web‐like chromatin structures called neutrophil extracellular traps (NETs) [11]. These structures are built from a DNA framework with granular proteins including histones, myeloperoxidase (MPO), and neutrophil elastase (NE) [11, 12]. Under normal conditions, NETs play a protective role by trapping and killing invading pathogens [13, 14, 15]. However, when inflammation persists, too much NETs extrusion—termed NETosis—liberates noxious mediators that damage alveolar epithelium and pulmonary microvascular endothelium. This disrupts the alveolar‐capillary barrier and makes lung injury worse [16, 17]. In addition, abnormal buildup of NETs may get in the way of normal lung repair processes [18]. Clinical studies have found high levels of NETs biomarkers in bronchoalveolar lavage fluid (BALF) and blood of preterm infants with BPD, and these levels correlate with how severe the disease is [19]. Animal models have also shown large amounts of NET deposits in LPS‐induced BPD lung tissue [19, 20]. These findings point to excessive NETosis as a critical pathological link connecting prenatal inflammation to postnatal lung development, whereas the underlying mechanisms still need to be worked out.

Extracellular vesicles derived from human umbilical cord mesenchymal stem cells (hUCMSCs), particularly microvesicles (MVs), act as key mediators of paracrine functions and demonstrate significant therapeutic potential in lung injury repair [21, 22]. Preclinical studies indicate that MVs protect against lung injury through multiple mechanisms, including reducing inflammation, promoting blood vessel growth, and modulating immune responses [23, 24]. Phase I clinical trials have provided initial evidence of the safety of using mesenchymal stem cells derived from human umbilical cords to treat BPD, with MVs considered central to their therapeutic efficacy [25, 26]. However, whether MVs alleviate BPD‐associated lung injury by modulating excessive NETosis formation remains unclear. Although in vitro experiments suggest MVs can directly inhibit NETosis, the in vivo regulatory mechanisms—potentially involving indirect pathways or multi‐signaling networks—warrant further investigation.

This work asked whether aberrant NET formation worsens BPD in rats given LPS before birth and whether MVs alleviate pulmonary injury via suppression of this pathway. Our findings may provide insights into the immunopathological mechanisms of BPD and establish an experimental foundation for MVs‐based acellular therapies.

## 2 Materials and Methods

### 2.1 Animals

Sprague‐Dawley (SD) rats obtained from the Laboratory Animal Center of Chongqing Medical University were maintained at a specific pathogen‐free facility of the Children's Hospital of Chongqing Medical University. The Animal Ethics Committee of the Children's Hospital of Chongqing Medical University approved all experimental protocols beforehand. Rats were housed under standard conditions (22 ± 1°C, 12 h light/dark cycle) with unrestricted access to food and water. Newborn pup survival was checked on a daily basis.

### 2.2 Induction of Prenatal BPD by Intra‐Amniotic LPS

Pregnant Sprague‐Dawley rats, 8–10 weeks old, received intra‐amniotic injections on gestational day 19.5 to establish the BPD model. We anesthetized the rats with inhaled isoflurane and made a midline abdominal incision to see the uterine horns. Starting near the left ovary, we injected amniotic sacs clockwise, with no more than 10 sacs per horn. To trigger prenatal inflammation, we injected either LPS (10 μg/50 μL normal saline (NS) per sac, *Escherichia coli* 055: B5, Sigma‐Aldrich, St. Louis, MO, USA) or an equal volume of NS (sham control) into the amniotic sac. Group assignment was random. Two days after the injection, we performed a cesarean section under anesthesia and delivered all pups from the injected sacs. Pups were then placed with foster mothers.

To assess the functional role of NETs to disease pathogenesis, we administered two distinct pharmacological NETosis inhibitors intranasally. Diphenyleneiodonium chloride (DPI) was given once daily during postnatal days 3–5 at 1 mg/kg. Sivelestat was given daily during postnatal days 3–10 at 5 mg/kg. We calculated doses for each pup individually and adjusted them according to body weight.

### 2.3 Purification and Verification

To prepare fetal bovine serum (FBS) depleted of exosomes, we spun it at 120,000 *g* for 12 hours at 4°C. hUCMSC‐conditioned medium (cells provided by the Chongqing Stem Cell Engineer Research Center) served as the source for MV purification. To generate MVs, we seeded 1 × 10^5^ cells (passages 4–7) onto each 100‐mm dish and let them grow for 48 hours in 10 mL of DMEM/F‐12 GBQ medium supplemented with 10% exosome‐depleted FBS. We removed cellular contaminants from the supernatant by spinning at low speeds at 4°C: 400 *g* for 5 minutes and then 1500 *g* for 10 minutes. We then spun the clarified supernatant at 18,000 *g* for 30 minutes at 4°C to collect the MVs fraction. After washing, we kept the purified MVs at −80°C. Their morphology and size distribution were assessed using transmission electron microscopy (TEM, Hitachi, HT‐7700, Tokyo, Japan) and nanoparticle tracking analysis (NTA, Particle Metrix PMX120, Munich, Germany), respectively.

### 2.4 MV Delivery and Specimen Collection

On postnatal day 7, we gave MVs (40 μL) into the windpipe to pups in the treatment group; controls received NS at the same volume. On postnatal day 14, we induced anesthesia by injecting pentobarbital sodium (40 mg/kg) intraperitoneally. We drew blood samples from the retro‐orbital plexus, spun them at 2500 rpm for 10 minutes at room temperature, and kept serum at −80°C. We then opened the chest and clamped the left main bronchus. We washed the right lung three times with 1.5 mL of ice‐cold phosphate‑buffered saline (PBS) to obtain BALF, then spun it at 2500 rpm for 10 min at 4°C; the supernatant was stored at −80°C. We snap‐froze the right lung, and fixed the left lung for microscopic examination.

### 2.5 Quantification of Alveolar Morphometry

Mid‐region transverse sections (4 μm) of the left lung were prepared and embedded in paraffin. Following hematoxylin–eosin staining, alveolar growth was quantified by radial alveolar count (RAC) and mean linear intercept (MLI) according to a previously established protocol [27]. All representative hematoxylin and eosin‐stained images presented in the figures include a 50 μm scale bar.

### 2.6 Isolation and Stimulation of Neutrophils

Rat bone marrow neutrophils were separated using Percoll density gradient centrifugation. Briefly, femurs and tibias of euthanized rats were harvested and flushed with ice‐cold RPMI 1640 medium containing antibiotics. The cell suspension obtained was passed through a 70 μm cell strainer and then centrifuged. The pellet was reconstituted in PBS and gently layered on top of a pre‐formed discontinuous Percoll gradient consisting of 2 mL of 80% Percoll, 1.5 mL of 65% Percoll, and 1.5 mL of 55% Percoll (bottom to top). After centrifugation at 1200 *g* for 30 min at 4°C, cells collected from the interface between the 65% and 80% Percoll layers were washed and treated with erythrocyte lysis buffer. Purified neutrophils were resuspended in RPMI 1640 supplemented with 10% FBS. For stimulation, neutrophils (1 × 10^6^ cells/mL) were treated with LPS (100 ng/mL) or phorbol 12‐myristate 13‐acetate (PMA, 50 nM, P1585, Sigma‐Aldrich, St. Louis, MO, USA) for 4 h at 37°C.

### 2.7 Immunofluorescence

Neutrophils were fixed with 4% paraformaldehyde (DF0135, LEAGENE, Beijing, China) and blocked with 5% bovine serum albumin (BSA, Biosharp). For lung tissues, paraffin‐embedded sections were first dewaxed, then subject to heat‐induced antigen retrieval in citrate buffer, and finally blocked with 5% (BSA, Biosharp, Hefei, Anhui, China). All samples were incubated overnight at 4°C with primary antibodies targeting citrullinated histone H3 (CitH3; 1:100, AF0009, Beyotime, Shanghai, China) and MPO (1:100, AG2657, Beyotime, Shanghai, China). The next day, we added appropriate fluorescence‐labeled secondary antibodies for 1 hour at room temperature (protected from light), followed by nuclear counterstaining with 4′,6‐diamidino‐2‐phenylindole (DAPI). To visualize NETs, isolated neutrophils were seeded onto poly‐L‐lysine‐coated slides, stimulated them as indicated, fixed them with 4% paraformaldehyde, and stained them with 1 μmol/L SYTOX Green (KGE2504‐500, KeyGEN BioTECH, Nanjing, Jiangsu, China) for 30 minues in the dark. A Leica laser confocal microscope (C2+ system, Nikon, Japan) was used to capture images. All images within the same experiment were acquired using identical laser power, gain, and offset settings. Each figure includes scale bars (50 μm).

### 2.8 Immunohistochemistry

We dewaxed paraffin sections, rehydrated them, and retrieved antigens in citrate buffer at pH 6.0. The activity of endogenous peroxidase was blocked, and sections were treated with 5% BSA. We incubated these sections overnight at 4°C with a rabbit anti‐MPO primary antibody (1:100, R25062, ZenBio, Chengdu, Sichuan, China). After washing, an horseradish peroxidase‐conjugated secondary antibody was applied, followed by development with the chromogenic substrate DAB. Nuclei were counterstained with hematoxylin. We examined the slides with a Carl Zeiss optical microscope and captured images at 400‐fold magnification. For each section, we selected three random nonoverlapping fields. A 50 μm scale bar appears in all images to indicate size. Microscope settings remained constant during image acquisition, which ensured comparability across samples.

### 2.9 Enzyme‐Linked Immunosorbent Assay (ELISA) Detection of Free Double‑Stranded DNA (dsDNA)

We measured free dsDNA in lung tissue homogenates and serum samples with the Quant‐iT PicoGreen dsDNA Detection Kit (P7589, Thermo Fisher Scientific, Waltham, MA, USA). The samples were diluted with TE buffer (a commonly used Tris‐EDTA buffer). The fluorescence was recorded at 480/520 nm using a Synergy HT microplate reader. Concentrations were determined from a standard curve that ranged from 250 pg/mL to 1 μg/mL.

### 2.10 Flow Cytometry

To assess neutrophil purity, we flushed bone marrow cells from femurs and tibiae. These cells were separated by Percoll gradient centrifugation. We stained the cells with 7‐AAD, anti‐Ly‐6G‐PE (1:200, BioLegend, San Diego, CA, USA), and anti‐CD11b‐APC (1:200, BioLegend, San Diego, CA, USA). For neutrophils from lung tissue, we minced the right upper lobe and digested it with type IV collagenase (collagenase IV, 2 mg/mL, C4‐BIOC, Sigma‑Aldrich, St. Louis, MO, USA) for 30 minutes at 37°C. The resulting cell suspension was filtered and stained with the same antibody cocktail. We performed flow cytometry on a BD FACSCanto Plus (BD Biosciences, Franklin Lakes, NJ, USA) and analyzed the data with FlowJo v10.8 (FlowJo LLC, Ashland, OR, USA). Neutrophils were identified as live cells with Ly‐6G^+^CD11b^+^ cells.

### 2.11 Western Blotting

Proteins were extracted separately from hUCMSCs and from MVs. These samples were resolved by sodium dodecyl sulfate‐polyacrylamide gel electrophoresis and then electrotransferred to polyvinylidene difluoride membranes. To probe the membranes, we used the following primary antibodies: rabbit anti‐TSG‐6 (1:1000, Proteintech, Rosemont, IL, USA), mouse anti‐Alix (1:1000, Proteintech, Rosemont, IL, USA), and mouse anti‐TSG101 (1:1000, Proteintech, Rosemont, IL, USA).

### 2.12 Statistical Analysis

All statistical analyses were conducted with GraphPad Prism 10.0 (GraphPad Software, San Diego, CA, USA). Quantitative data that came from at least three independent experiments or biological replicates are expressed as mean ± standard error of the mean (SEM). Exact *n* values are provided in the figure legends. To compare multiple groups, we used one‐way or two‐way ANOVA as appropriate, followed by post hoc tests. A *p*‐value below 0.05 was considered statistically significant (*p < 0.05, **p < 0.01, ***p < 0.001, and ****p < 0.0001). For the in vivo therapeutic experiments, we benchmarked the efficacy of hUCMSC‐MVs against two NETosis inhibitors (diphenyleneiodonium chloride and sivelestat).

## 3 Results

The exact values supporting the figures are presented in Supporting Information S1.

### 3.1 Purification and Phenotypic Analysis of hUCMSC‐MVs

We used three methods to check whether we had successfully isolated hUCMSC‐MVs (Figure 1). First, transmission electron microscopy (TEM) revealed MVs with the expected bilayer membrane structure (Figure 1A). Second, immunoblotting verified that MVs carried typical markers (TSG101 and CD63) and had no cellular protein contamination (Figure 1B). Third, nanoparticle tracking analysis (NTA) revealed a mix of particle size, mostly between 100 and 900 nm, with most particles coming in at about 177.4 nm (Figure 1C). These results confirm the successful isolation and typical characteristics of hUCMSC‐MVs.

**FIGURE 1:**
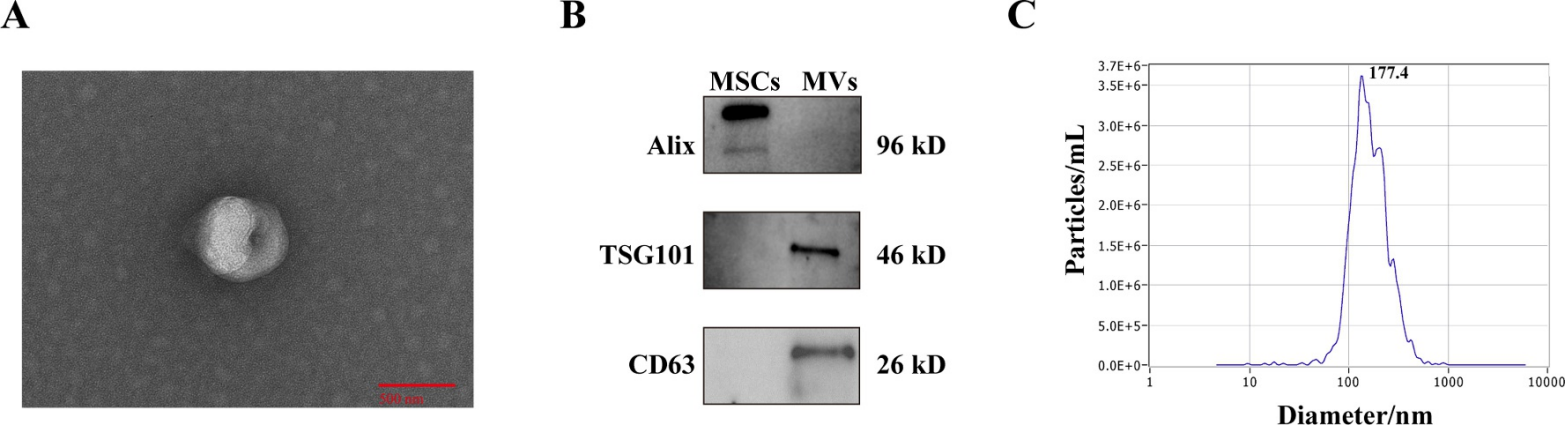
Characterization of microvesicles (MVs). MVs exhibit characteristic morphology, biomarker expression, and size distribution. (A) Transmission electron microscopy reveals the typical cup‐shaped morphology of MVs. Scale bar, 500 nm. (B) Western blot confirms the specific protein profile of isolated MVs, enriched in vesicle markers (e.g., CD63 and TSG101) and devoid of cytoplasmic contaminants (Alix). (C) Nanoparticle tracking analysis shows a homogeneous population with a mean diameter ± standard deviation of 177.4 ± 25 nm, consistent with the expected size range of MVs. *n* = 2 independent isolations.

### 3.2 LPS Exposure Before Birth Produces BPD‐Like Alveolar Changes

To model BPD in preterm rats, we injected LPS into the amniotic sac and delivered the pups by cesarean section delivery (Figure 2A). At postnatal day 14, LPS‐exposed pups exhibited severe alveolar simplification: the MLI was significantly increased, and the RAC was decreased compared to controls (Figure 2B). Tracheal administration of hUCMSC‐MVs at postnatal day 7 markedly attenuated these abnormalities (Figure 2C,D). MLI decreased and RAC increased in MVs‐treated pups (Figure 2C,D). These results confirm that this model recapitulates key features of BPD and is responsive to MV‐based intervention.

**FIGURE 2:**
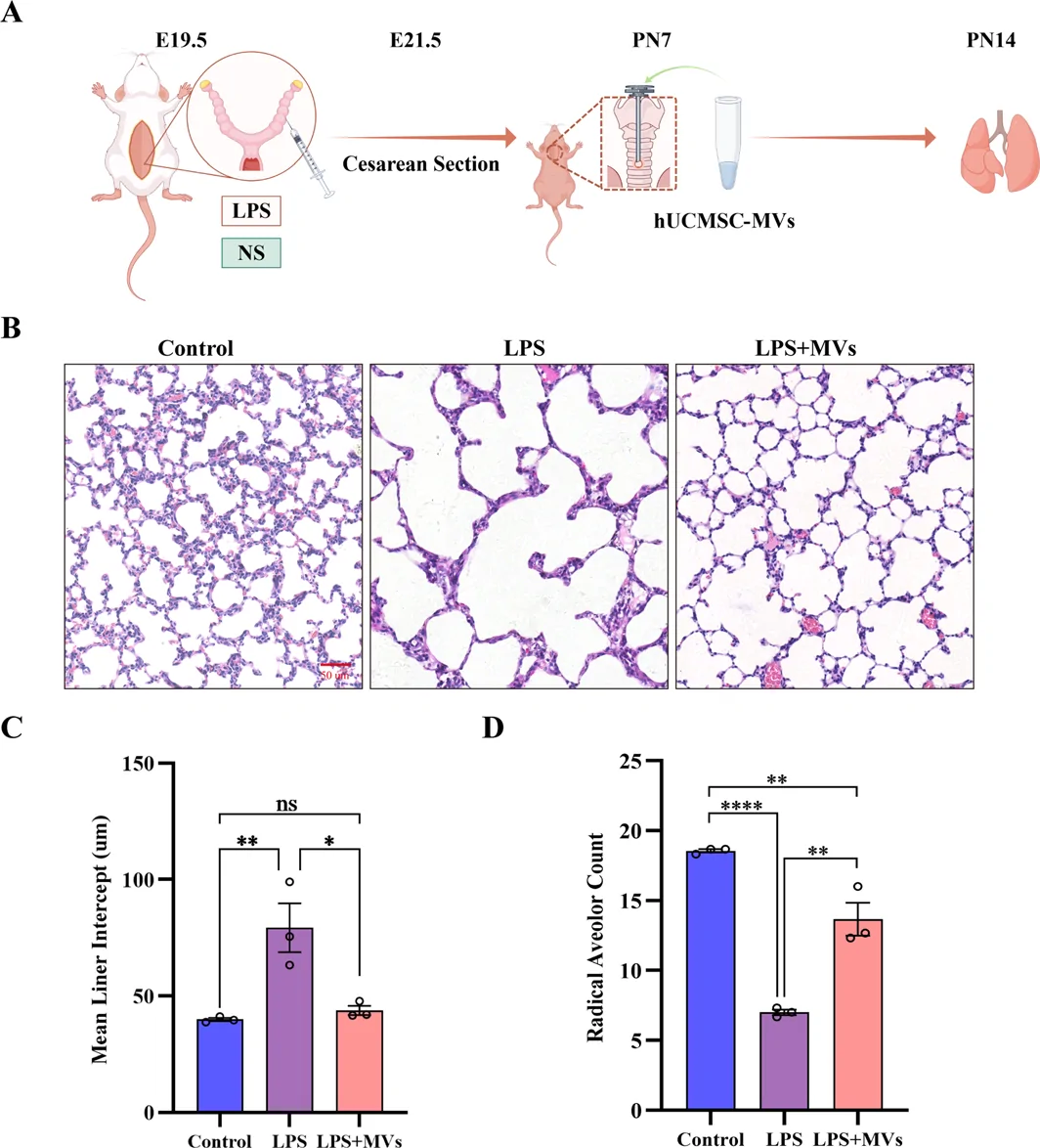
Prenatal lipopolysaccharide (LPS) exposure successfully models bronchopulmonary dysplasia (BPD)‐like alveolar simplification in preterm rats. (A) Schematic of intra‐amniotic LPS injection protocol. (B) Representative hematoxylin and eosin‐stained lung sections at postnatal day 14 demonstrate enlarged and simplified alveoli in the LPS group compared to controls. Scale bar, 50 μm. (C, D) Morphometric analyses confirm significant alveolar destruction, evidenced by mean linear intercept and radial alveolar count. (*n* = 3 animals per group). Data are mean ± standard error of the mean. E, embryonic day; MV, microvesicles; NS, normal saline; ns, not significant; PN, postnatal day; *p < 0.05, **p < 0.01, ****p < 0.0001.

### 3.3 Excessive NETs Formation in BPD Lungs Is Attenuated by hUCMSC‐MVs

Having observed alveolar simplification, we next investigated the role of NETs formation. Immunofluorescence staining of LPS‐exposed pups revealed significant colocalization of citrullinated histone H3 (CitH3) and MPO, indicating structural NET formation (Figure 3A,B). Supporting this finding, immunohistochemistry confirmed marked MPO‐positive neutrophil infiltration in the alveolar spaces (Figure 3C,D). The NET burden extended beyond the lungs. ELISA showed significantly elevated levels of dsDNA in both BALF and serum of BPD group (Figure 3E,F), confirming that excessive NETosis was both a local and systemic phenomenon. Prenatal LPS exposure left neutrophils in a primed state. We found this by stimulating bone marrow neutrophils from LPS‐exposed animals with PMA outside the body: they released more NETs than controls did (Figure S1A). Quantification of these results confirmed the enhanced NETs release in the BPD group (Figure 3G).

**FIGURE 3:**
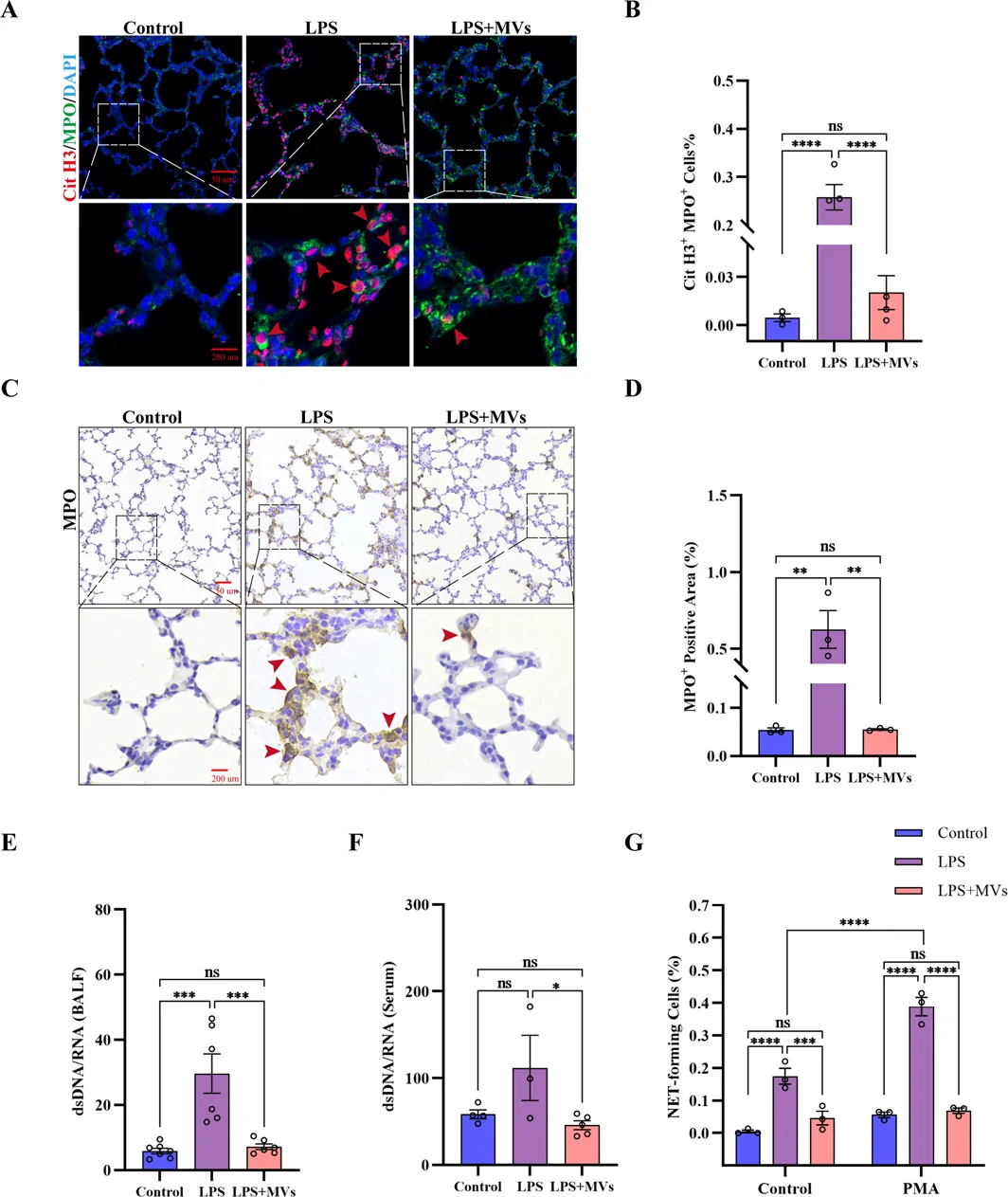
Microvesicle (MV) treatment attenuates excessive neutrophil extracellular traps (NETs) formation in bronchopulmonary dysplasia (BPD) lungs. (A, B) Immunofluorescence staining reveals pronounced co‐localization (red arrow) of citrullinated histone H3 (CitH3, red) and myeloperoxidase (MPO, green) in BPD lungs, indicating in situ NET formation. This signal is diminished in the MV group. Scale bar, 50 μm. *n* = 3 animals per group. (C, D) MPO immunohistochemistry confirms extensive neutrophil infiltration in BPD lungs, which is alleviated by MVs. *n* = 3 animals per group. (E, F) MVs reduce the elevated NET burden in both bronchoalveolar lavage fluid (BALF) and serum. Double‐stranded DNA levels were quantified by enzyme‐linked immunosorbent assay. Analysis of BALF samples from biologically independent animals showed a significant increase in the BPD group (control, *n* = 7; lipopolysaccharide [LPS], *n* = 6), which was reduced after MV treatment (LPS + MVs, *n* = 6). A consistent trend was observed in serum samples from independent animals (control, *n* = 4; LPS, *n* = 3; LPS + MVs, *n* = 5). (G) Quantification of SYTOX Green fluorescence intensity in bone marrow‐derived neutrophils isolated from the indicated groups and stimulated with phorbol 12‐myristate 13‐acetate (PMA, 50 nmol/L) for 4 hours. The propensity for NET release in LPS‐primed neutrophils is suppressed by MVs treatment in vivo. Representative images are shown in Figure S1A. (*n* = 3 animals per group). Data are presented as mean ± standard error of mean. ns, not significant; *p < 0.05, **p < 0.01, ***p < 0.001, and ****p < 0.0001.

Intratracheal administration of hUCMSC‐MVs reversed these abnormalities. The treatment reduced CitH3/MPO co‐localization in lungs, lowered dsDNA levels in BALF and serum, and suppressed the enhanced NETs release from primed neutrophils (Figure 3A–G; Figure S1A). These findings demonstrate that hUCMSC‐MVs effectively attenuate the excessive NETosis seen in the BPD lung.

### 3.4 hUCMSC‐MVs Directly Suppress LPS‐Induced NETosis in Isolated Neutrophils

To determine whether hUCMSC‐MVs act directly on neutrophils, we isolated highly pure bone marrow neutrophils (Figure 4A,B). We pretreated cells with hUCMSC‐MVs before adding NETosis inducers (LPS, PMA, or LPS + PMA). Compared to untreated controls, pretreatment with MVs reduced NETs formation under all stimulation conditions. Higher magnification imaging clearly delineated the characteristic web‐like DNA structures decorated with CitH3 and MPO, the defining features of classical NETs (Figure 4C). Quantitative analysis confirmed the pattern: MV pretreatment significantly lowered NET formation regardless of which inducer was used (Figure 4D–F). Thus, hUCMSC‐MVs can intrinsically inhibit the NETosis process in neutrophils.

**FIGURE 4:**
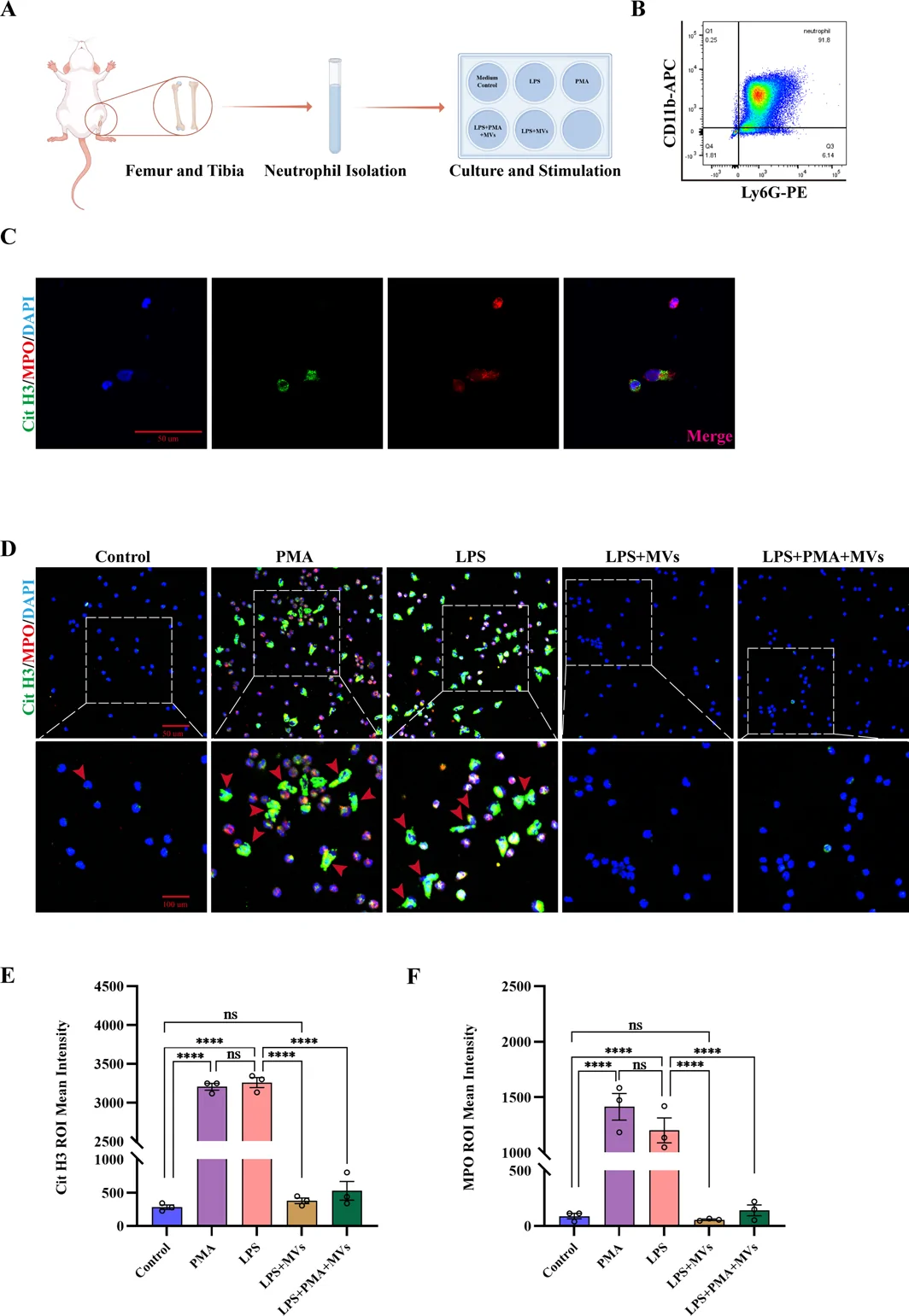
Microvesicles (MVs) directly inhibit neutrophil extracellular traps (NETs) formation in isolated neutrophils. (A, B) Schematic and experimental design for evaluating NETs formation (NETosis) inhibition. Bone marrow‐derived neutrophils were isolated, and their high purity was verified by flow cytometry (B). To assess the inhibitory capacity of MVs, cells were assigned to five treatment groups as depicted: Control, phorbol 12‐myristate 13‐acetate (PMA), lipopolysaccharide (LPS), LPS + MVs, and LPS + PMA + MVs. (C) High‐magnification imaging of NET structures. Representative immunofluorescence images of bone marrow‐derived neutrophils stimulated with PMA (50 nmol/L) for 4 hours. Cells were stained for citrullinated histone H3 (CitH3, red) and myeloperoxidase (MPO, green). Nuclei were counterstained with 4′,6‐diamidino‐2‐phenylindole (DAPI, blue). Red arrows indicate typical NET structures characterized by extracellular DNA fibers decorated with CitH3 and MPO. Scale bar, 50 μm. (D–F) MVs directly suppress the NETosis processes induced by PMA and LPS in vitro. Representative immunofluorescence images (D) demonstrate the co‐localization (indicated by red arrows) of CitH3 (green) and MPO (red). Scale bars, 50 μm. Quantitative analyses of the mean fluorescence intensity for CitH3 (E) and MPO (F) confirm that both LPS and PMA individually induce robust NETosis. Notably, the addition of MVs significantly reduced NETosis signals in groups treated with LPS (compare LPS vs. LPS + MVs, and LPS + PMA vs. LPS + PMA + MVs), suggesting a modulatory effect on LPS‐mediated pathways (*n* = 3 independent neutrophil isolations per group). Data are mean ± SEM. ns, not significant; ****p < 0.0001.

### 3.5 hUCMSC‐MVs Ameliorate BPD by Suppressing NETosis In Vivo

We benchmarked MV treatment against two pharmacological NETosis inhibitors (diphenyleneiodonium chloride and sivelestat) in vivo. At postnatal day 14, LPS‐exposed lungs showed marked accumulation Ly6G^+^CD11b^+^ neutrophils. MV treatment significantly reduced this accumulation, and the reduction was comparable to that achieved with the NETosis inhibitors (Figure 5A,B). This attenuated neutrophil influx was accompanied by improved alveolar architecture. Both MV‐treated and inhibitor‐treated pups showed decreased MLI and increased RAC compared to untreated BPD animals (Figure 5C–E). At the molecular level, the same pattern held. hUCMSC‐MV treatment and NETosis inhibitor treatment both produced a significant reduction in CitH3/MPO colocalization and MPO‐positive area (Figure 5F–I). The convergence of therapeutic effects between hUCMSC‐MVs and direct NETosis inhibitors points to a common mechanism: inhibition of excessive NETosis. NETosis suppression represents a key pathway through which hUCMSC‐MVs confer lung protection in BPD.

**FIGURE 5:**
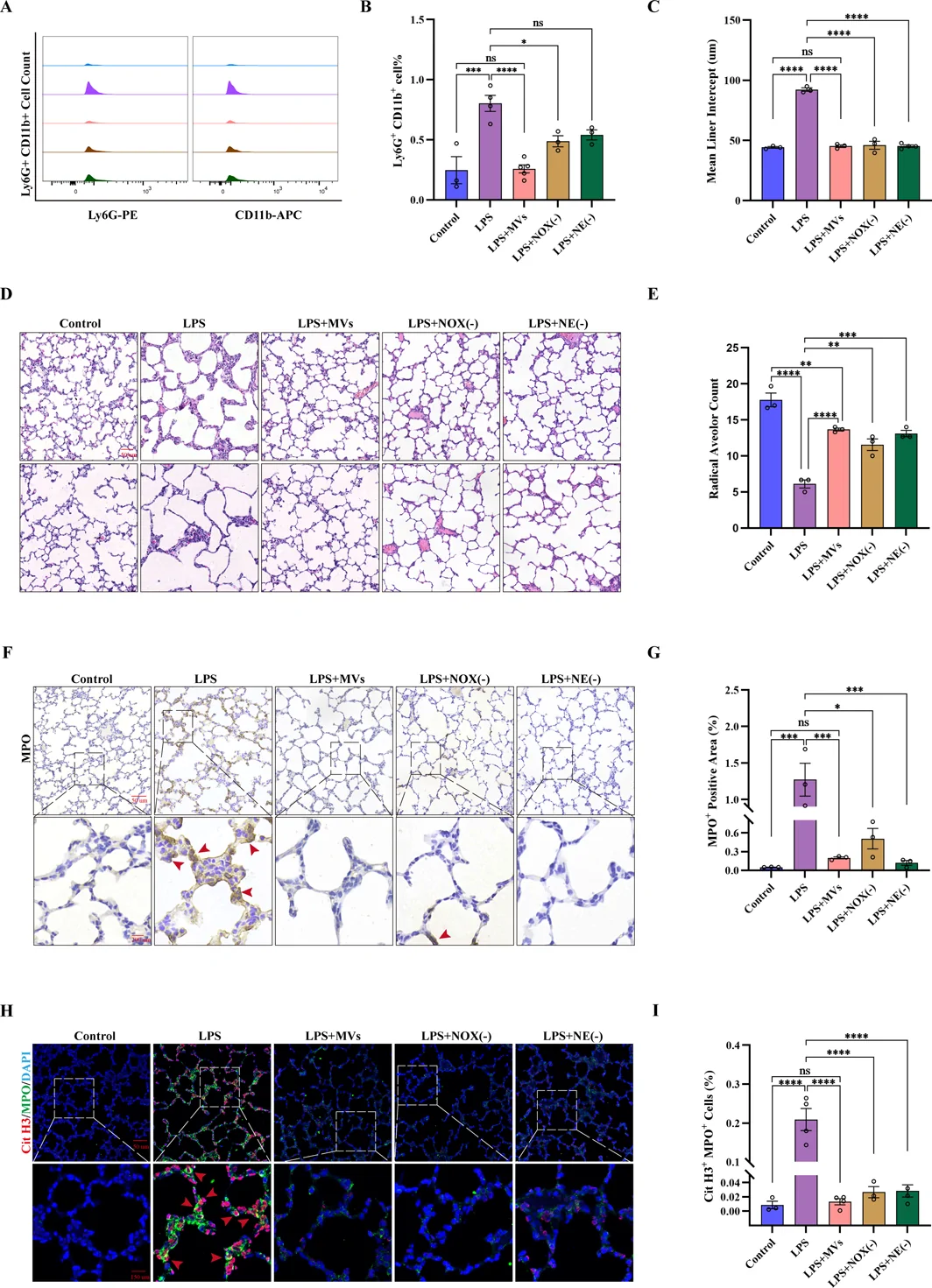
Therapeutic effects of microvesicles (MVs) in bronchopulmonary dysplasia (BPD) involve reduction of neutrophil extracellular traps (NETs) and amelioration of alveolar injury. (A, B) MVs reduce neutrophil accumulation in BPD lungs. Flow cytometric analysis of lung homogenates shows a significant increase in the proportion of Ly6G^+^CD11b^+^ neutrophils in the lipopolysaccharide (LPS)‐induced BPD model. This increase was significantly attenuated by treatment with either MVs or a pharmacological NETs formation inhibitor. Data are derived from the following numbers of biologically independent animals per group: Control (*n* = 3), LPS (*n* = 4), LPS + MVs (*n* = 5), LPS + NETosis inhibitor (*n* = 3). (C–E) Improvement of alveolar structure. Therapeutic intervention with MVs or the NETosis inhibitors ameliorated LPS‐induced alveolar simplification, as evidenced by a decreased mean linear intercept (C) and an increased radial alveolar count (E). Representative hematoxylin and eosin‐stained lung sections are shown in (D). Scale bar, 50 μm (*n* = 3 animals per group). (F, G) Reduction of global neutrophil infiltration. Myeloperoxidase (MPO) immunohistochemistry and quantification show extensive neutrophil accumulation in BPD lungs, which was significantly mitigated by both MV and NETosis inhibitor treatments (*n* = 3 animals per group). (H, I) Specific suppression of NETosis. Correspondingly, immunofluorescence analysis reveals that the proportion of citrullinated histone H3 (CitH3) and MPO double‐positive cells (indicative of active NET‐forming neutrophils) is markedly increased in the BPD model and significantly reduced by the treatments. Data are from biologically independent animals with the following sample sizes per group: Control (*n* = 3), LPS (*n* = 4), LPS + MVs (*n* = 4), LPS + NADPH oxidase inhibitor (*n* = 3), and LPS + neutrophil elastase inhibitor (*n* = 3). Data are presented as mean ± standard error of the mean. ns, not significant; *p < 0.05, **p < 0.01, ***p < 0.001, ****p < 0.0001.

## 4 Discussion

Our data are consistent with prior clinical and experimental observations: the accumulation of abnormal NETs represents a critical pathological event in the LPS‐induced preterm BPD rat model [19, 20]. We applied three methods to detect NETs in the lungs of preterm BPD rats: histology, in situ structural validation, and systemic biomarker measurements. All of them showed NETs deposition at different perspectives. Prenatal LPS exposure left neutrophils in a preactivated state. These neutrophils became more likely to release NETs once they encountered an inflammatory microenvironment. This observation may point to a possible priming effect. Prenatal inflammatory exposure may make neutrophils hyperresponsive to later stimuli as BPD progresses. The consequence is excessive NETs formation, which shifts NETosis from a physiological defense mechanism toward pathological injury. Excessive NETs deposition exerts dual deleterious effects: it directly compromises the structural integrity of both alveolar epithelial cells and vascular endothelium, while also promoting alveolar‐capillary barrier disruption via the liberation of cytotoxic mediators including histones and MPO [16, 17, 18]. Such injury may further compromise the lung's intrinsic capacity for tissue repair.

In addition to confirming that NETosis drives BPD, this work offers new mechanistic insight: excessive NETosis bridges prenatal inflammation with postnatal lung injury. hUCMSC‐MVs alleviate this damage by suppressing NET formation, performing as effectively as known NETosis inhibitors. However, the precise molecular mechanisms by which hUCMSC‐MVs suppress NETosis remain to be fully elucidated. Based on available literature, we propose the following focused and testable mechanistic hypotheses to clarify whether MV‐mediated inhibition occurs through surface ligand‐receptor interactions or through modulation of redox signaling pathways.

One possibility is that MV‐mediated inhibition may involve surface ligand‐receptor interactions. Mesenchymal stem cell‐derived extracellular vesicles carry various adhesion molecules on their surface, including CD44 and ICAM‐1, which can mediate specific binding and internalization of vesicles by neutrophils [28]. Among these, CD44, as the primary receptor for hyaluronan, plays an important role in neutrophil activation and migration [29]. Previous reports have demonstrated that mesenchymal stem cell‑derived extracellular vesicles (MSC‐EVs) exert protective effects on tissues by binding to injured cells via surface CD44 [30]. Toll‐like receptor 4 (TLR4) is a key upstream molecule in LPS‐induced neutrophil activation, and evidence suggests that MSC‐EVs can suppress inflammatory responses by modulating TLR4 signaling [31, 32]. Therefore, we hypothesize that hUCMSC‐MVs may interact with neutrophils through CD44‐ or TLR4‐mediated receptor recognition, thereby initiating downstream inhibitory signals [33, 34].

A second mechanism, involving the regulation of redox signaling pathways, may also be key. Nicotinamide adenine dinucleotide phosphate (NADPH) oxidase 2 (NOX2)‐dependent reactive oxygen species (ROS) generation is a central driver of NETosis [35]. Recent studies have demonstrated that MSC‐EVs suppress NETosis by modulating the ROS/AKT pathway, thereby alleviating sepsis‐induced acute lung injury [36]. Other studies have confirmed that human umbilical cord mesenchymal stem cell‐derived extracellular vesicles transfer functional mitochondria to neutrophils, restoring mitochondrial function and reducing mitochondrial ROS levels, thereby significantly decreasing NETs formation in ischemia‐reperfusion injury [37, 38]. Given that LPS‐induced NETosis depends on a burst of ROS, we speculate that hUCMSC‐MVs may interrupt the NETosis cascade through several mechanisms: inhibition of NOX2 activity, scavenging of intracellular ROS, or restoration of mitochondrial function.

Notably, the aforementioned pathways are not mutually independent: surface receptor recognition may trigger downstream signaling cascades that subsequently regulate NOX2 activity and ROS generation. Therefore, we propose that hUCMSC‐MVs may exert their protective effects through a hierarchical mechanism in which receptor recognition regulates ROS production and subsequently inhibits NETosis. This hypothesis brings together surface interactions and redox regulation, thereby offering a clear direction for future studies.

In addition, the limitations of this model should be taken into account when interpreting these findings. Although the preterm rat model is widely employed, it incompletely mirrors the complex multifactorial nature of human BPD—a condition caused by a combination of prolonged exposure to hyperoxia, ventilator support, and infections acquired after birth [9]. Animal models have taught us a great understanding about BPD pathophysiology. However, as Ambalavanan points out, each model have inherent limitations in replicating the complex clinical scenario of preterm infants [39]. One major limitation is the difference between species. Rodents and humans differ in lung development, immune maturation, and inflammatory responses which create inherent challenges for translating findings from animals to humans [40, 41]. Before hUCMSC‐MVs can be used clinically, several therapeutic challenges must be addressed. First, the route of administration is critical. Preclinical studies have shown that, compared with intravenous injection, endotracheal administration is more effective in alleviating neonatal hyperoxic lung injury, likely due to enhanced pulmonary bioavailability [42, 43]. Second, the effective dose may depend on the chosen route. Evidence suggests that aerosolized inhalation can achieve therapeutic efficacy at lower doses than intravenous infusion [44]. Third, the timing of treatment needs careful consideration. Clinical data indicate that earlier intervention, when given within the first 30 days of life, is associated with better outcomes in preterm infants with BPD [26]. Future preclinical studies should systematically evaluate these variables: dosing regimens, treatment windows, and routes of administration [45, 46, 47]. They should also include long‐term assessments of pulmonary function and neurodevelopmental outcomes. Such long‐term assessments are important because BPD survivors often have persistent pulmonary function deficits extending into adolescence and adulthood, and children born extremely preterm face higher risks of cognitive and motor impairments that may persist throughout childhood and beyond [46, 47]. If MV‐based therapies are to be translated into clinical practice, preclinical models must address these long‐term outcomes. A comprehensive understanding of lasting benefits and safety profile is essential before moving forward.

Our findings indicate that hUCMSC‐MVs alleviate prenatal LPS‐triggered BPD‐like pulmonary damage in preterm rats through suppression of aberrant NETosis. Based on the available literature, we propose two focused mechanistic hypotheses: hUCMSC‐MVs may interact with neutrophils through CD44/TLR4‐mediated receptor recognition, subsequently regulating NOX2/ROS‐dependent redox signaling pathways, and ultimately inhibiting NETs formation. This hierarchical framework, in which receptor recognition regulates ROS production and subsequently inhibits NETosis, provides clearly testable directions for future research. It should be noted that the protein kinase C/extracellular signal‑regulate/NOX2 axis, as a potential upstream regulatory pathway of NETosis, remains speculative and similarly requires systematic validation as a testable hypothesis in future studies. Future investigations should employ genetic tools, pharmacological inhibitors, and live‐cell imaging techniques to systematically validate the aforementioned molecular events and their relative contributions in BPD therapy. Uncovering how hUCMSC‐MVs modulate NETosis will both advance our knowledge of BPD's immunopathology and lay the groundwork for designing NETosis‐targeted therapies.

## 5 Conclusion

In this prenatal LPS‐induced BPD model, inflammatory overactivation was shown to prime neutrophils and drive excessive NETosis. This process shifts from host defense to pathological injury. hUCMSC‐MVs attenuated BPD‐like alveolar simplification as effectively as established NETosis inhibitors. These findings have two main implications: they give us a better understanding of the role NETs play in BPD pathogenesis and provide a theoretical foundation for developing MV‐based therapies that target NETosis.

## Author Contributions

Yi Lu and Wen Tan: experimental operations, data collection, data analysis, and manuscript writing. Xiao Yang and Tingting Luo: animal care and maintenance. Chenghao Mei: software analysis. Chao Gong, Jinhua Ma, and Li Zhong: manuscript review and revision. Ou Zhou: technical guidance and manuscript review. Daiyin Tian: project management, funding provision, manuscript review, and proofreading. All authors have read and approved the final manuscript.

## Funding

This research was supported by the National Natural Science Foundation of China (Grants 82300002 and 82171712), Natural Science Foundation of Chongqing, China (Grants CSTB2023NSCQ‐BHX0051 and CSTB2022NSCQ‐MSX0822), and Program for Youth Innovation in Future Medicine, Chongqing Medical University (Grant W0063).

## Ethics Statement

Animal experiments were approved by the Animal Ethics Committee of Children's Hospital of Chongqing Medical University (Approval No. CHCMU‐IACUC20231012005) and followed institutional guidelines for laboratory animal care and use.

## Conflicts of Interest

The authors declare no conflicts of interest.

## Supporting information


Supporting Information S1

**Figure S1:** Representative images of phorbol 12‐myristate 13‐acetate (PMA)‐induced neutrophil extracellular traps (NETs) release from neutrophils. (A) Bone marrow‐derived neutrophils were isolated from the indicated groups (Control, lipopolysaccharide [LPS], LPS+ microvesicles [MVs]) and stimulated with PMA (50 nmol/L) for 4 hours. Extracellular DNA release was visualized by SYTOX Green staining. Scale bar, 50 µm. Quantification of fluorescence intensity is shown in Figure 3G. *n* = 3 animals per group.

## Data Availability

The numerical data supporting the findings of this study are provided in Supporting Information S1, including the raw values for all bar graphs and scatter plots presented in Figures 1, 2, 3, 4, 5 and Figure S1. Additional raw data can be obtained from the corresponding author upon reasonable request.
