# Supplementary material for: Human Umbilical Cord Mesenchymal Stem Cell‐Derived Microvesicles Alleviate Bronchopulmonary Dysplasia in Preterm Rats by Inhibiting Neutrophil Extracellular Trap Formation
Source: Pediatr Discov. 2026 Aug 5;4(3):e70065. doi: 10.1002/pdi3.70065 (PMC13443229; doi:10.1002/pdi3.70065)
Supplement: Supplementary file 1 [file PDI3-4-e70065-s001.docx]

**S1A.**

**Supplemental Figure S1. Representative images of phorbol 12-myristate 13-acetate (PMA)-induced neutrophil extracellular traps (NETs) release from neutrophils.** (A) Bone marrow-derived neutrophils were isolated from the indicated groups (Control, lipopolysaccharide [LPS], LPS+ microvesicles [MVs]) and stimulated with PMA (50 nmol/L) for 4 hours. Extracellular DNA release was visualized by SYTOX Green staining. Scale bar, 50 µm. Quantification of fluorescence intensity is shown in Figure 3G. *n*=3 animals per group.
